# Lactic acid as a major contributor to hand surface infection barrier and its association with morbidity to infectious disease

**DOI:** 10.1038/s41598-021-98042-4

**Published:** 2021-09-20

**Authors:** Yuki Nishioka, Kenichi Nagano, Yoshitaka Koga, Yasuhiro Okada, Ichiro Mori, Atsuko Hayase, Takuya Mori, Kenji Manabe

**Affiliations:** 1 Personal Health Care Products Research, Kao Corporation, 2-1-3, Bunka, Sumida-ku, Tokyo, 131-8501 Japan; 2 Biological Science Laboratories, Kao Corporation, 2606 Akabane, Ichikai, Haga, Tochigi, 321-3497 Japan; 3 Analytical Science Laboratories, Kao Corporation, 2606 Akabane, Ichikai, Haga, Tochigi, 321-3497 Japan

**Keywords:** Microbiology, Medical research

## Abstract

Although the surface of the human hands contains high antimicrobial activity, studies investigating the precise components involved and the relationship between natural antimicrobial activity and morbidity in infectious diseases are limited. In this study, we developed a method to quantitatively measure the antimicrobial activity of hand surface components. Using a clinical survey, we validated the feasibility of our method and identified antimicrobial factors on the surface of the human hand. In a retrospective observational study, we compared the medical histories of the participants to assess infectious diseases. We found that the antimicrobial activity on the surface of the hands was significantly lower in the high morbidity group (N = 55) than in the low morbidity group (N = 54), indicating a positive association with the history of infection in individuals. A comprehensive analysis of the hand surface components indicated that organic acids, especially lactic acid and antimicrobial peptides, are highly correlated with antimicrobial activity. Moreover, the application of lactic acid using the amount present on the surface of the hand significantly improved the antimicrobial activity. These findings suggest that hand hygiene must be improved to enhance natural antimicrobial activity on the surface of the hands.

## Introduction

Contact infection is spread from one person to another by contaminated “hands.” The hand is an important route in the transmission of pathogens through direct physical contact with infected individuals or by indirect contact through contaminated surfaces. Hand hygiene is one of the most effective ways to prevent the transmission of disease-causing microorganisms^1,2^. Transient microorganisms, including bacteria and viruses, on the outer layers of human skin are commonly associated with infection. These microorganisms can be transmitted through direct contact with the skin and removed by handwashing^2^. Although there are a number of related studies in the medical literature and specific hand hygiene-related guidelines in many countries that highlight the preventive effect of hand hygiene on infectious diseases, its implementation remains a challenge in healthcare institutions and the community^2,3^. Handwashing with soap or the use of alcohol-based and non-alcohol-based hand sanitizers with alcohol are common hand hygiene practices^4^. However, existing hand hygiene practices are unable to fully prevent contact infections because of the high frequency of self-inoculation and the high stability of pathogens in the environment^5,6^.

Moreover, maintaining hand hygiene using conventional methods in a cohort with low hygiene awareness, such as elementary school students, is difficult^4^. Under these conditions, leave-on-hand hygiene products with benzalkonium chloride are more likely to be effective in preventing infection than volatile alcohol-based hand sanitizers. However, the excessive use of antimicrobial agents poses a risk for the development of resistant bacteria and removes the lipid-based barrier, thus causing the loss of moisture^7,8^.

Lipid, protein, peptides, and low molecular weight substances on the surface of human hands naturally possess some levels of antimicrobial activity^9–13^. Thus, we focused on strengthening the surface infection barrier on the hands using the innate antimicrobial defense mechanism of the skin. Identifying the molecules involved in the inherent antimicrobial ability of the skin to maintain hand hygiene could promote the development of new natural antimicrobial agents with fewer side effects. Several publications have proposed that the skin infection barrier includes peptides that exert antimicrobial activity and initiate the host response, resulting in the release of cytokines, inflammation, angiogenesis, and re-epithelialization^14^. Other components present on the skin, such as lipids and organic acids, may also have antibacterial properties; however, their contribution to endogenous defenses remains poorly understood^15^. Studies investigating the effects of the activity of the hand surface infection barrier on the morbidity of infectious diseases are lacking. Since pathogens do not typically enter the host through the intact epithelium of the hand, transmission not only involves passing the pathogen from one hand to another, but also conveying the pathogen on the hand to a suitable portal of entry, such as the mucosal tissues of the mouth, nose, or eyes^16^. Once the pathogenic microorganisms enter the mouth and nose after passing through the skin surface barrier, natural defense mechanisms such as saliva, oral mucosal moisture, nasal mucociliary clearance, or natural killer cell activity in peripheral bold mononuclear cells (PBMNCs) play crucial roles in reducing the infection risk^17–19^. Thus, investigating the relationship between these factors and susceptibility to infection is important.

We developed a method to evaluate the inherent antimicrobial activity of the skin, which was then compared with the medical history of the participants to assess for infectious diseases in order to clarify the importance of hand antimicrobial activity. Furthermore, we identified the crucial components of this barrier in combination with the quantification of organic acids, amino acids, proteins, and lipids present on the surface of the hands to promote the development of new natural antimicrobial technology.

## Results

### Preliminary study to characterize hand antimicrobial activity using in vivo and in vitro methods

**Figure 1:**
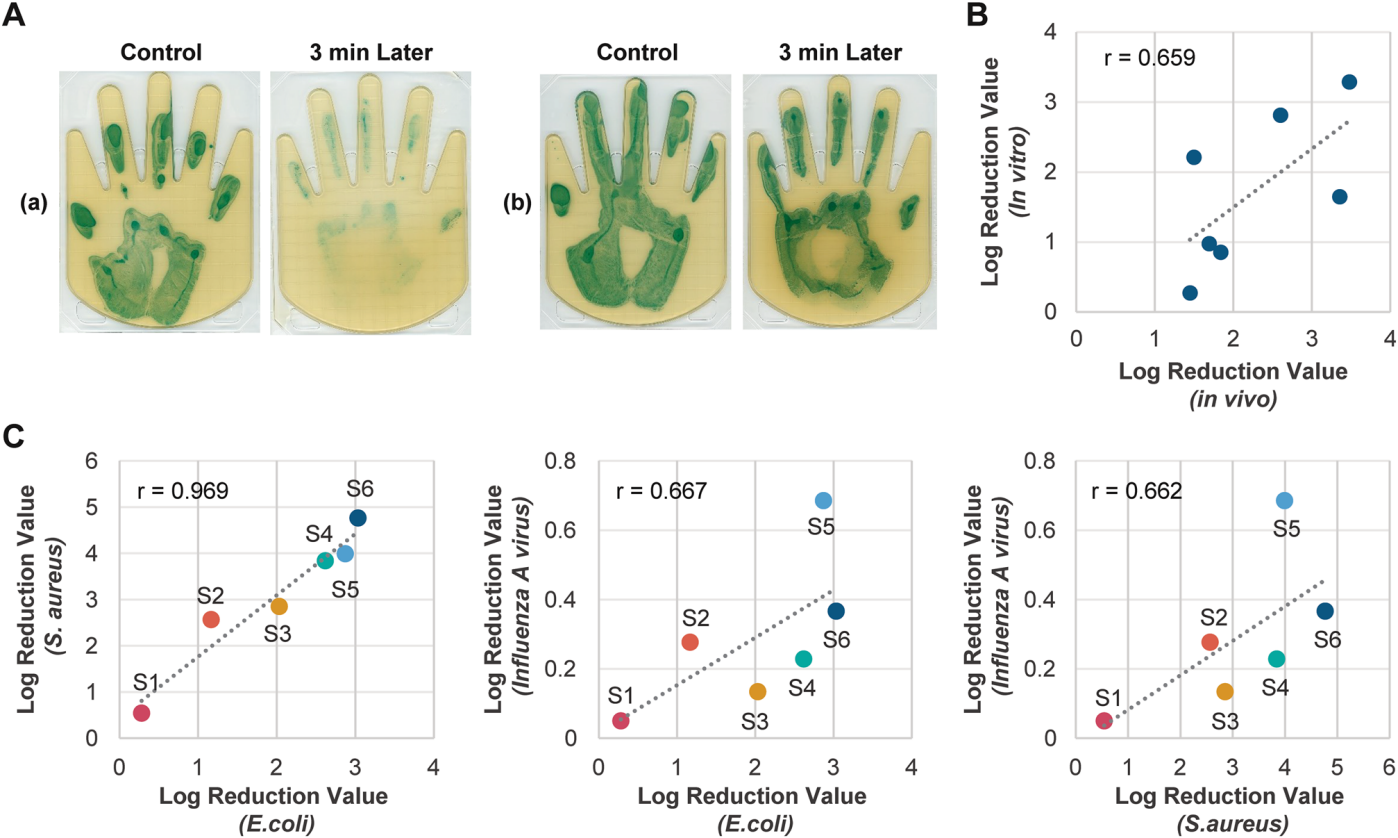
Individual differences in hand antimicrobial activity and the importance of surface components. (**A**) Qualitative comparison results using agar medium for (a) hands with high antimicrobial activity and (b) hands with low antimicrobial activity. Results for 30 s (control) and 3 min after applying *E. coli* (OD = 0.2) on the hand. (**B**) Correlation between the antimicrobial activity on the hands (in vivo) and the antimicrobial activity of the surface components on the hands (in vitro). The antimicrobial activity on the hands was measured by applying an *E. coli* solution (OD = 0.2) for 3 min. The surface components of the hands were collected with a cotton swab, and concentrated to 0.8 μL/cm^2^ using DMSO. The antimicrobial activity was measured by mixing the sample with equal amounts of *E. coli* (OD = 0.02) and by allowing it to produce a reaction for 30 min. The log reduction value denotes the relative logarithmic reduction of viable bacteria. The value of Pearson’s correlation coefficient is indicated in the graph. (**C**) Correlation of the antimicrobial activity of the surface components on the hands against *E. coli*, *S. aureus*, and influenza A virus. Symbols with the same color indicate individual participants (S1–S6) between the three graphs. *E. coli*, *S. aureus*, and influenza A virus were evaluated using undiluted samples, eightfold diluted samples, and 100-fold diluted samples, respectively, due to the toxicity of DMSO. The log reduction value denotes the relative logarithmic reduction of the bacterial or viral number. The value of Pearson’s correlation coefficient is indicated in each graph.

As a pilot study, we examined various methods for measuring antimicrobial activity found on the hands. No blue colonies were observed in the imprints of hands not immersed in an *Escherichia coli* solution, suggesting that the microorganisms that formed the blue colonies were not found in the normal skin microbiota (data not shown). As shown in Fig. 1A, in study sample (a), the number of bacteria decreased remarkably within 3 min compared with that in the control (30 s), indicating the ability of the human hand to inactivate *E. coli* over time. In contrast, in study sample (b), no significant difference was observed between 30 s (control) and 3 min, indicating individual differences in the antimicrobial ability of the human hands. In addition, the phenomenon of *E. coli* inactivation observed in subject (a) was not observed immediately after washing hands, suggesting that the existing hand surface components are a contributing factor (Supplementary Fig. S1).

Figure 1B shows the correlation of the log reduction rate against *E. coli *in vivo and the in vitro method in seven participants, indicating a positive correlation (r = 0.66, *P* = 0.1; Pearson’s correlation test). The hand surface components were important factors in defining the hand surface infection barrier (Supplementary Fig. S2). These findings indicate that this in vitro method is appropriate for evaluating the function of this infection barrier. Figure 1C shows the antimicrobial activity of the hand surface components in six participants (S1–S6) against *E. coli* and *Staphylococcus aureus*, as well as antiviral activity against influenza A virus. Undiluted, eightfold diluted, and 100-fold diluted solutions of extracted samples against *E. coli*, *S. aureus*, and influenza A virus were used. As a result, positive correlations (r = 0.970 and *P* = 0.001 [*E. coli* vs. *S. aureus*], r = 0.667 and *P* = 0.147 [*E. coli* vs. influenza A virus], and r = 0.662 and *P* = 0.152 [*S. aureus* vs. influenza A virus] ) were observed, using Pearson’s correlation test. These results suggest that the hand surface components can act on a wide range of microbial species. However, considering the differences in the dilution rate, the susceptibility of the hand surface components was one order lower against *E. coli* than against *S. aureus* and influenza virus.

### Relationship between antimicrobial activity on the surface of hands and morbidity to infection

**Figure 2:**
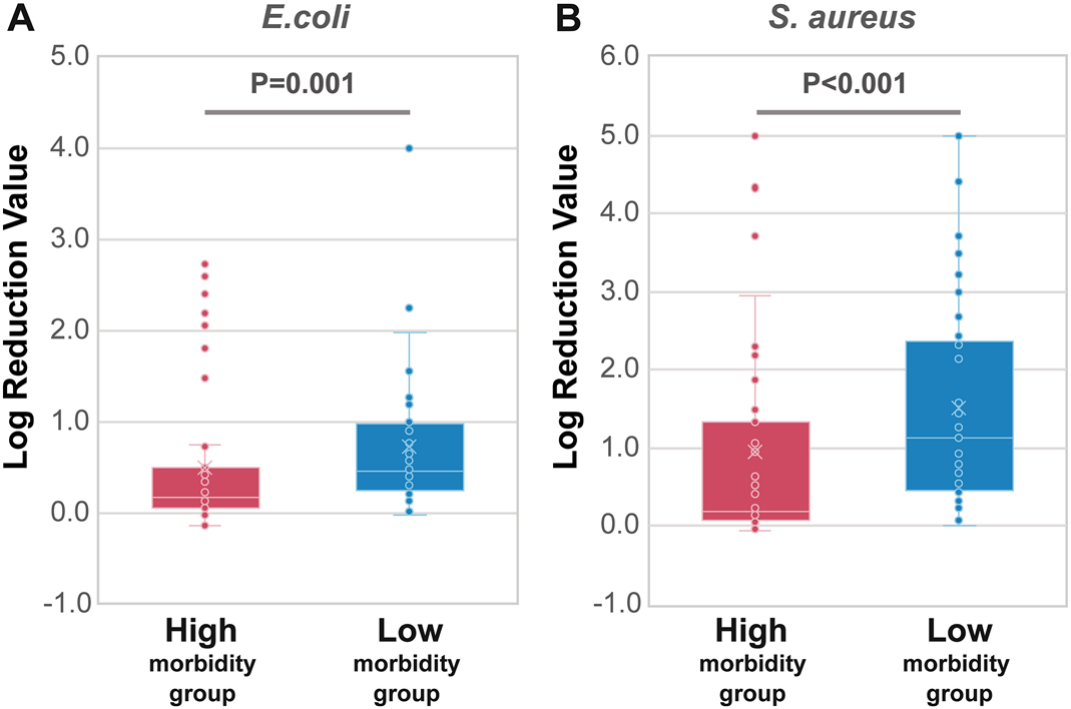
Relationship between antimicrobial activity and morbidity to infection. Result of antimicrobial activity against *E. coli* and *S. aureus*. Left (red) and right (blue) denote the high morbidity group (N = 55) and the low morbidity group (N = 54), respectively. Antimicrobial activity was measured using an in vitro method, where the log reduction value denotes the relative logarithmic reduction of viable bacteria. The two groups were compared using the Mann–Whitney U test.

Next, we assessed the influence of the antimicrobial activity of the hands on morbidity and infection in participants with similar lifestyles (Supplementary Tables S1 and S2; Supplementary Fig. S3). Figure 2 shows the antimicrobial activity of the hand surface components of each group against *E. coli* (Fig. 2A) and *S. aureus* (Fig. 2B). The antimicrobial activity against *E. coli* was significantly lower in the high morbidity group than in the low morbidity group (median of low morbidity group: 0.466; median of high morbidity group: 0.159; *P* = 0.001). Similar results were observed for antimicrobial activity against *S. aureus* (median of low morbidity group: 1.137; median of high morbidity group: 0.191; *P* < 0.001). In addition, our study showed that in addition to antimicrobial activity on the hands, oral mucosal moisture was also associated with infection prevalence (*P* = 0.006) (Table 1). In contrast, no difference was found between the groups in terms of NK cell activity and saliva volume (Table 1). These results were not related to hygiene behaviors, such as hand hygiene, mask, and vaccination frequency, since the frequency of these behaviors was higher in the high morbidity group than in the low morbidity group (Table S2).

**Table 1:** Physiological functions in a retrospective study investigating the relationship between hand antimicrobial activity and morbidity to infection. The data were first tested for normality using the Shapiro–Wilk test. Data showing normality were assessed for variability using the f-test and t-test, in accordance with variability. Non-normal data were tested using the Mann–Whitney U test.

	Low morbidity group			High morbidity groupSS			*P*-value^2^	
	n^1^	Mean	SD	n^1^	Mean	SD		
Body temperature (°C)	54	37.0	0.3	55	37.0	0.3	0.666	
Saliva volume (g)	54	2.4	1.5	55	2	1.4	0.153	
Oral mucosal moisture (%)	54	30	3.3	55	27.8	4.4	0.006	
NK cells activity in PBMCs (%)	54	49.2	18.4	55	50.7	18	0.756	
Nasal mucociliary clearance (s)	48	700	339	42	638	307	0.371	
Hand antimicrobial activity (log reduction)								
For *E. coli*	54	0.7	0.8	55	0.5	0.7	0.001	
For *S. aureus*	54	1.5	1.3	55	0.9	1.5	< 0.001	

^1^Parameters for which the number of samples is lower than all participants indicate the parameters for which the corresponding number of samples was not determined.

^2^*P*-values were calculated using the Mann–Whitney U test.

**Figure 3:**
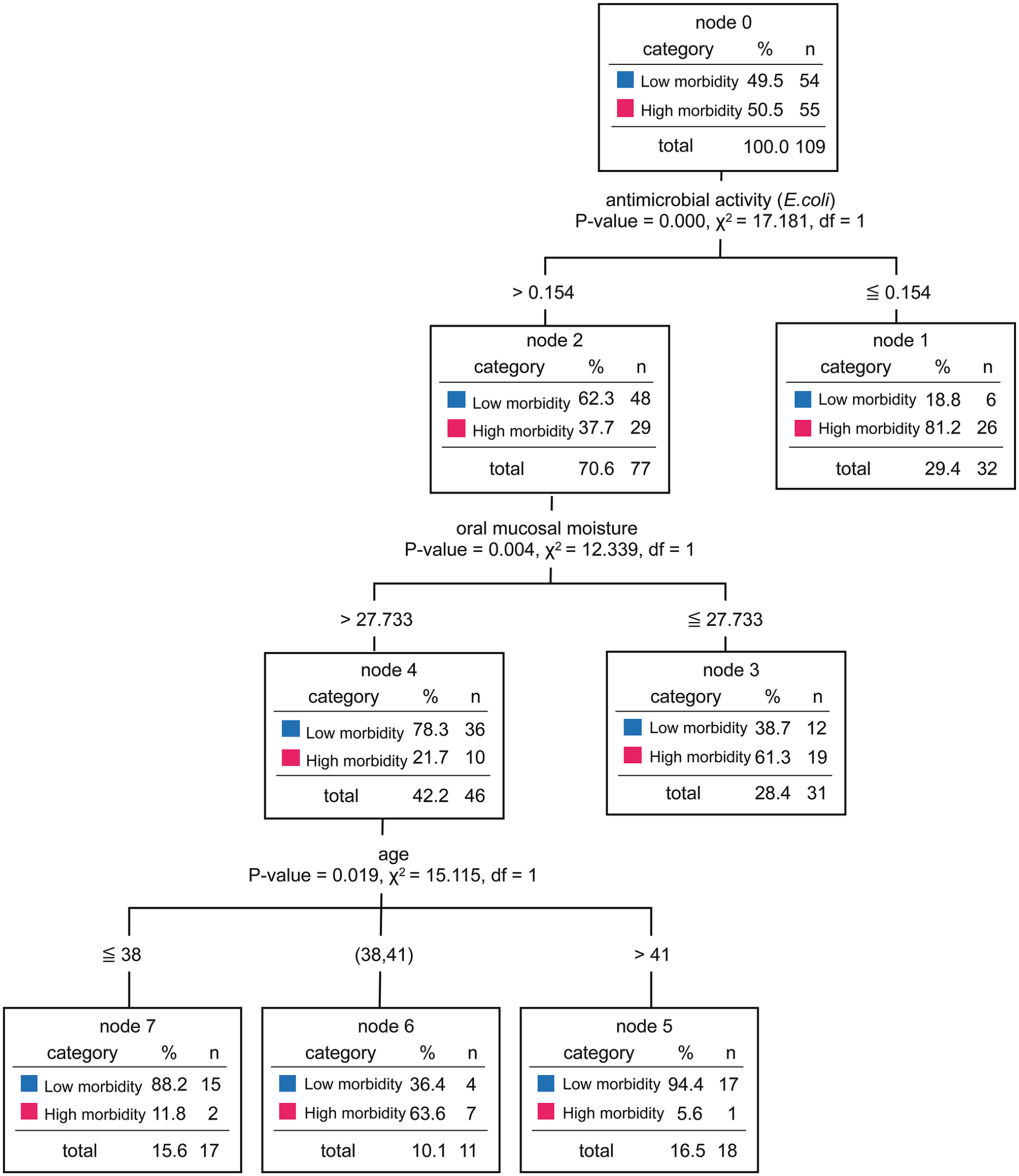
Decision tree analysis to assess the biological parameters and physiological functions. The biological parameters (age, sex, and body temperature) and physiological functions (hand antimicrobial activity, saliva volume, oral mucosal moisture, NK cells activity in PBMCs, and nasal mucociliary clearance) were used. The odds ratios (ORs) and 95% confidence interval were obtained using the cross-tabulation method.

To evaluate the importance of parameters associated with infectious disease morbidity, we performed a hierarchical decision tree analysis, containing all the measured biological parameters (Fig. 3). The tree comprised a total of eight nodes and five terminal nodes (nodes that had no child nodes). The hand antimicrobial activity (*E. coli*) was found to be closely accompanying the morbidity to infection, indicating a possible causal association between them or presence of a third factor influencing both dermal antimicrobial activity and morbidity. The odds ratio (OR) based on the reference value (log reduction value for *E. coli*: 0.154) calculated in the decision tree analysis was 7.2 times greater (95% confidence interval [CI], 2.64–19.50). For participants with a log reduction value for *E. coli* of 0.154 or higher, the OR based on the reference value of mucosal moisture was 5.7 times greater (95% CI, 2.08–15.60). With regard to age, cross-tabulation based on the 41- and 38-year age groups showed that the results were significant in the 41-year age group. However, the 95% CI ranged from 0.01 to 1.08, indicating that the reliability of the results was low. These results are detailed in Table S3.

### Identification of antimicrobial components on hands using comprehensive analysis

Additionally, we conducted another clinical study to identify the factors involved in hand antimicrobial activity. In this survey, we analyzed the hand surface components and calculated their correlation with antimicrobial activity against *E. coli*, *S. aureus*, and influenza A virus (Supplementary Fig. S4). The components analyzed included organic acids, amino acids, lipids, and some antimicrobial proteins. In terms of the amounts found on the hand surface, lactic acid was prominently present among all compounds (Tables 2, 3, and 4). Small amounts of lipids were found on the hands. Among them, fatty acids were relatively abundant, among which C16: 0, C14: 0, and C12: 0 were abundantly detected (Table 4). Antimicrobial peptide-related compounds on the hands were analyzed using shotgun proteomics, and dermcidin, prolactin-inducible protein, and lysozyme C were detected (Table 4). Next, we analyzed the correlation between antimicrobial activity and surface components using Holm-corrected Pearson’s correlation coefficients, as shown in Tables 2, 3 and 4. Bold numbers denote significant data (*P* < 0.05), while compounds in bold indicate compounds that were significantly correlated with all antimicrobial activities. With regard to organic acids, lactic acid was found to have a significant correlation with all microbial species (Table 2, Supplementary Fig. S5). In addition to lactic acid, malic acid, 2-hydroxybutyric acid, citric acid, ascorbic acid, azelaic acid, 2-oxoglutarate, suberic acid, and fumaric acid showed a high positive correlation with antimicrobial activity. In the amino acid group, alanine, creatinine, taurine, choline, creatine, and p-aminobenzoic acid were highly correlated with antimicrobial activity (Table 3). Subsequently, free fatty acids (FFA), wax ester (WE), cholesteryl ester (ChE), squalene (SQ), squalene epoxide (squalene epoxide), squalene monohydroperoxide (SQOOH), diacylglycerol (DAG), and triacylglycerol were analyzed. However, none had a significant positive correlation with the antimicrobial activity of hand surface components, and only had a negative correlation with DAG. Lastly, among the antimicrobial proteins, prolactin-inducible protein was significantly correlated with antimicrobial activity.

**Table 2:** Relationship between hand surface organic acids and antimicrobial activity.

	Components^3^	Value^1^			Correlation coefficient^2^			*P*-value^2^		
		Maximum	Minimum	Mean	*E. coli*	*S. aureus*	*Influenza A Virus*	*E. coli*	*S. aureus*	*Influenza A virus*
Organic acid (nmol/cm^2^)	**Lactic acid**	**906**	**6**	**212**	**0.5561**	**0.6836**	**0.6689**	**0.000**	**0.000**	**0.000**
	Urocanic acid	14.90	0.17	3.25	**0.3080**	0.2514	0.1816	**0.023**	0.067	0.189
	**Malic acid**	**1.308**	**0.150**	**0.585**	**0.5834**	**0.6339**	**0.6272**	**0.000**	**0.000**	**0.000**
	Asparagine	1.710	0.062	0.570	0.0487	0.0611	0.1931	0.726	0.661	0.162
	**2-hydroxybutyricacid**	**0.223**	**0.029**	**0.116**	**0.5170**	**0.6253**	**0.6325**	**0.000**	**0.000**	**0.000**
	Glycolic acid	0.2140	0.0144	0.0652	0.0841	− 0.0392	− 0.0738	0.545	0.778	0.596
	**Citric acid**	**0.2138**	**0.0027**	**0.0487**	**0.5335**	**0.5568**	**0.5353**	**0.000**	**0.000**	**0.000**
	**Ascorbic acid**	**0.1167**	**0.0000**	**0.0270**	**0.5750**	**0.6948**	**0.6946**	**0.000**	**0.000**	**0.000**
	**Azelaic acid**	**0.1136**	**0.0023**	**0.0203**	**0.4100**	**0.3013**	**0.3391**	**0.002**	**0.027**	**0.012**
	**2-oxoglutarate**	**0.0698**	**0.0000**	**0.0202**	**0.5981**	**0.7455**	**0.7537**	**0.000**	**0.000**	**0.000**
	**Suberic acid**	**0.0426**	**0.0016**	**0.0127**	**0.3889**	**0.2933**	**0.3232**	**0.004**	**0.031**	**0.017**
	Succinic acid	0.1403	0.0006	0.0113	− 0.0095	0.1295	0.0238	0.946	0.351	0.864
	**Fumaric acid**	**0.0375**	**0.0000**	**0.0105**	**0.5234**	**0.7057**	**0.6669**	**0.000**	**0.000**	**0.000**
	Sebacic acid	0.01209	0.00008	0.00161	0.1328	0.2002	0.1262	0.339	0.147	0.363
	Pyridoxine	0.024862	0.000000	0.000845	− 0.0890	− 0.0661	− 0.0938	0.522	0.635	0.500
	Nicotinic acid	0.002420	0.000000	0.000257	0.0638	0.0492	0.0635	0.647	0.724	0.648
	Pantothenic acid	0.0019567	0.0000000	0.0000642	− 0.0262	− 0.0580	− 0.0776	0.851	0.677	0.577
	FFA [C12:0]	0.23	0.01	0.04	− 0.2586	− 0.2410	− 0.1374	0.059	0.079	0.322
	SQ	0.18	0.01	0.04	− 0.0874	− 0.0706	− 0.0919	0.530	0.612	0.509
	FFA[C18:0]	0.15	0.01	0.03	− **0.2934**	− **0.3120**	− 0.2542	**0.031**	**0.022**	0.064
	FFA[C18:1]	0.10	0.00	0.02	0.0688	− 0.0405	0.0740	0.621	0.771	0.595
	WE	0.10	0.00	0.01	− 0.2548	− 0.2342	− 0.2209	0.063	0.088	0.109
	FFA[C16:1]	0.06	0.00	0.01	0.0188	− 0.1093	− 0.0826	0.893	0.432	0.553
	SQepo	0.05	0.00	0.01	− 0.0573	0.0238	− 0.0126	0.681	0.864	0.928
	FFA[C15:0]	0.03	0.00	0.01	− 0.0093	− 0.1336	− 0.1445	0.947	0.335	0.297
	ChE	0.020	0.000	0.005	0.0221	0.0224	0.0261	0.874	0.872	0.851
	**DAG**	**0.030**	**0.000**	**0.004**	− **0.2956**	− **0.3269**	** − 0.3575**	**0.030**	**0.016**	**0.008**
	SQOOH	0.010	0.000	0.001	− 0.0947	− 0.0545	− 0.2466	0.496	0.695	0.072

^1^The numbers correspond to the amounts that exist on the hand surface and are arranged in order of mean value.

^2^Correlation and *P*-value between each compound and antimicrobial activity were calculated using Holm-corrected Pearson’s correlation coefficients. Bold numbers show significant differences (*P*-value less than 0.05).

^3^Bold indicates the compounds that were significantly correlated with all antibacterial and antiviral activities.

**Table 3:** Relationship between hand surface amino acids and antimicrobial activity.

	Components^3^	Value^1^			Correlation coefficient^2^			*P*-value^2^		
		Maximum	Minimum	Mean	*E. coli*	*S. aureus*	*Influenza A virus*	*E. coli*	*S. aureus*	*Influenza A virus*
Amino acid (nmol/cm^2^)	Serine	53	1	18	0.2210	0.2003	0.1883	0.108	0.146	0.173
	Glycine	33	1	12	0.2161	0.1950	0.2063	0.117	0.158	0.134
	**Alanine**	**11.2**	**0.3**	**4.2**	**0.3305**	**0.3134**	**0.2961**	**0.015**	**0.021**	**0.030**
	Ornithine	14.4	0.1	3.4	− 0.1398	** − 0.2774**	− 0.2370	0.314	**0.042**	0.084
	Threonine	8.3	0.3	3.1	0.1930	0.1564	0.1532	0.162	0.259	0.269
	Pyroglutamic acid	5.7	0.5	2.6	0.2458	0.2263	0.2500	0.073	0.100	0.068
	Aspartic acid	7.5	0.2	2.3	0.1044	0.0872	0.1288	0.452	0.531	0.353
	Valine	7.7	0.1	1.8	0.1422	0.0986	0.1655	0.305	0.478	0.232
	Proline	4.3	0.2	1.7	0.0711	− 0.0097	0.0188	0.609	0.945	0.893
	Leucine	3.8	0.1	1.5	0.2086	0.1871	0.2667	0.130	0.175	0.051
	Histidine	5.4	0.1	1.4	− 0.0442	− 0.1346	− 0.1292	0.751	0.332	0.352
	Isoleucine	2.7	0.1	1.4	0.2340	0.2241	**0.2921**	0.089	0.103	**0.032**
	Tyrosine	2.38	0.02	0.86	0.1571	0.1038	0.1342	0.257	0.455	0.333
	Lysine	2.47	0.07	0.80	− 0.0441	− 0.1526	− 0.1133	0.751	0.271	0.415
	Phenylalanine	2.07	0.03	0.79	0.1926	0.1703	0.2416	0.163	0.218	0.078
	Glutamic acid	1.43	0.07	0.50	0.1637	0.1392	0.1938	0.237	0.316	0.160
	Arginine	2.88	0.03	0.22	0.1072	0.0915	0.0976	0.441	0.511	0.482
	**Creatinine**	**0.68**	**0.03**	**0.21**	**0.4436**	**0.5107**	**0.4907**	**0.001**	**0.000**	**0.000**
	Tryptophan	0.62	0.01	0.19	**0.2870**	0.2385	0.2443	**0.035**	0.082	0.075
	**Taurine**	**0.54**	**0.02**	**0.17**	**0.4780**	**0.5491**	**0.4796**	**0.000**	**0.000**	**0.000**
	Methionine	0.47	0.01	0.15	0.2076	0.1733	**0.3055**	0.132	0.210	0.025
	Citrulline	1.26	0.00	0.13	0.0808	0.0029	− 0.0742	0.561	0.984	0.594
	**Choline**	**0.274**	**0.014**	**0.092**	**0.3986**	**0.4507**	**0.4518**	**0.003**	**0.001**	**0.001**
	Xanthine	0.083	0.001	0.031	0.1577	0.2513	**0.2818**	0.255	0.067	0.039
	**Creatine**	**0.060**	**0.002**	**0.026**	**0.4442**	**0.4391**	**0.4126**	**0.001**	**0.001**	**0.002**
	Uric acid	0.094	0.000	0.022	− 0.1869	** − 0.3912**	** − 0.4526**	0.176	**0.003**	**0.001**
	**Carnitine**	**0.0145**	**0.0009**	**0.0058**	**0.4661**	**0.4594**	**0.4257**	**0.000**	**0.000**	**0.001**
	Cystine	0.0190	0.0002	0.0058	0.2032	0.1808	0.2010	0.141	0.191	0.145
	Tyramine	0.01071	0.00000	0.00062	0.1585	0.0731	0.2147	0.252	0.599	0.119
	**p-aminobenzoic acid**	**0.000397**	**0.000000**	**0.000074**	**0.4637**	**0.3999**	**0.3039**	**0.000**	**0.003**	**0.025**
	Inosine	0.000397	0.000000	0.000028	− 0.0376	− 0.1101	− 0.1011	0.787	0.428	0.467

^1^The numbers correspond to the amounts that exist on the hand surface and are arranged in order of mean value.

^2^Correlation and *P*-value between each compound and antimicrobial activity were calculated using the Holm-corrected Pearson’s correlation coefficients. Bold numbers show significant differences (*P*-value less than 0.05).

^3^Bold indicates the compounds that were significantly correlated with all antibacterial and antiviral activities.

**Table 4:** Relationship of hand surface lipids and proteins with antimicrobial activity.

	Components^3^	Value^1^			Correlation coefficient^2^			*P*-value^2^		
		Maximum	Minimum	Mean	*E. coli*	*S. aureus*	*Influenza A virus*	*E. coli*	*S. aureus*	*Influenza A virus*
Lipid (µg/cm^2^)	FFA [C16:0]	0.20	0.02	0.07	− 0.1985	− 0.1849	− 0.1729	0.150	0.181	0.211
	FFA [C14:0]	1.01	0.00	0.06	− 0.1633	− 0.1392	− 0.0382	0.238	0.316	0.784
	TAG	0.34	0.00	0.05	− 0.1961	− 0.1308	− 0.2086	0.155	0.346	0.130
	FFA [C12:0]	0.23	0.01	0.04	− 0.2586	− 0.2410	− 0.1374	0.059	0.079	0.322
	SQ	0.18	0.01	0.04	− 0.0874	− 0.0706	− 0.0919	0.530	0.612	0.509
	FFA [C18:0]	0.15	0.01	0.03	** − 0.2934**	** − 0.3120**	− 0.2542	**0.031**	**0.022**	0.064
	FFA [C18:1]	0.10	0.00	0.02	0.0688	− 0.0405	0.0740	0.621	0.771	0.595
	WE	0.10	0.00	0.01	− 0.2548	− 0.2342	− 0.2209	0.063	0.088	0.109
	FFA [C16:1]	0.06	0.00	0.01	0.0188	− 0.1093	− 0.0826	0.893	0.432	0.553
	SQepo	0.05	0.00	0.01	− 0.0573	0.0238	− 0.0126	0.681	0.864	0.928
	FFA [C15:0]	0.03	0.00	0.01	− 0.0093	− 0.1336	− 0.1445	0.947	0.335	0.297
	ChE	0.020	0.000	0.005	0.0221	0.0224	0.0261	0.874	0.872	0.851
	**DAG**	**0.030**	**0.000**	**0.004**	** − 0.2956**	** − 0.3269**	** − 0.3575**	**0.030**	**0.016**	**0.008**
	SQOOH	0.010	0.000	0.001	− 0.0947	− 0.0545	− 0.2466	0.496	0.695	0.072
Protein (emPAI)	Dermcidin	113	1	28	0.2139	**0.3793**	**0.3379**	0.120	**0.005**	**0.012**
	**Prolactin-inducible protein**	**4.2**	**0.0**	**1.4**	**0.3179**	**0.3862**	**0.3093**	**0.019**	**0.004**	**0.023**
	Lysozyme C	1.5	0.0	0.3	− 0.0090	− 0.0957	− 0.1007	0.948	0.491	0.469

^1^The numbers correspond to the amounts that exist on the hand surface and are arranged in order of mean value.

^2^Correlation and P-value between each compound and antimicrobial activity were calculated using Holm-corrected Pearson’s correlation coefficients. Bold numbers show significant differences (*P*-value less than 0.05).

^3^Bold indicates the compounds that were significantly correlated with all antibacterial and antiviral activities.

### Verification of the effect of lactic acid application on the hand antimicrobial activity

**Figure 4:**
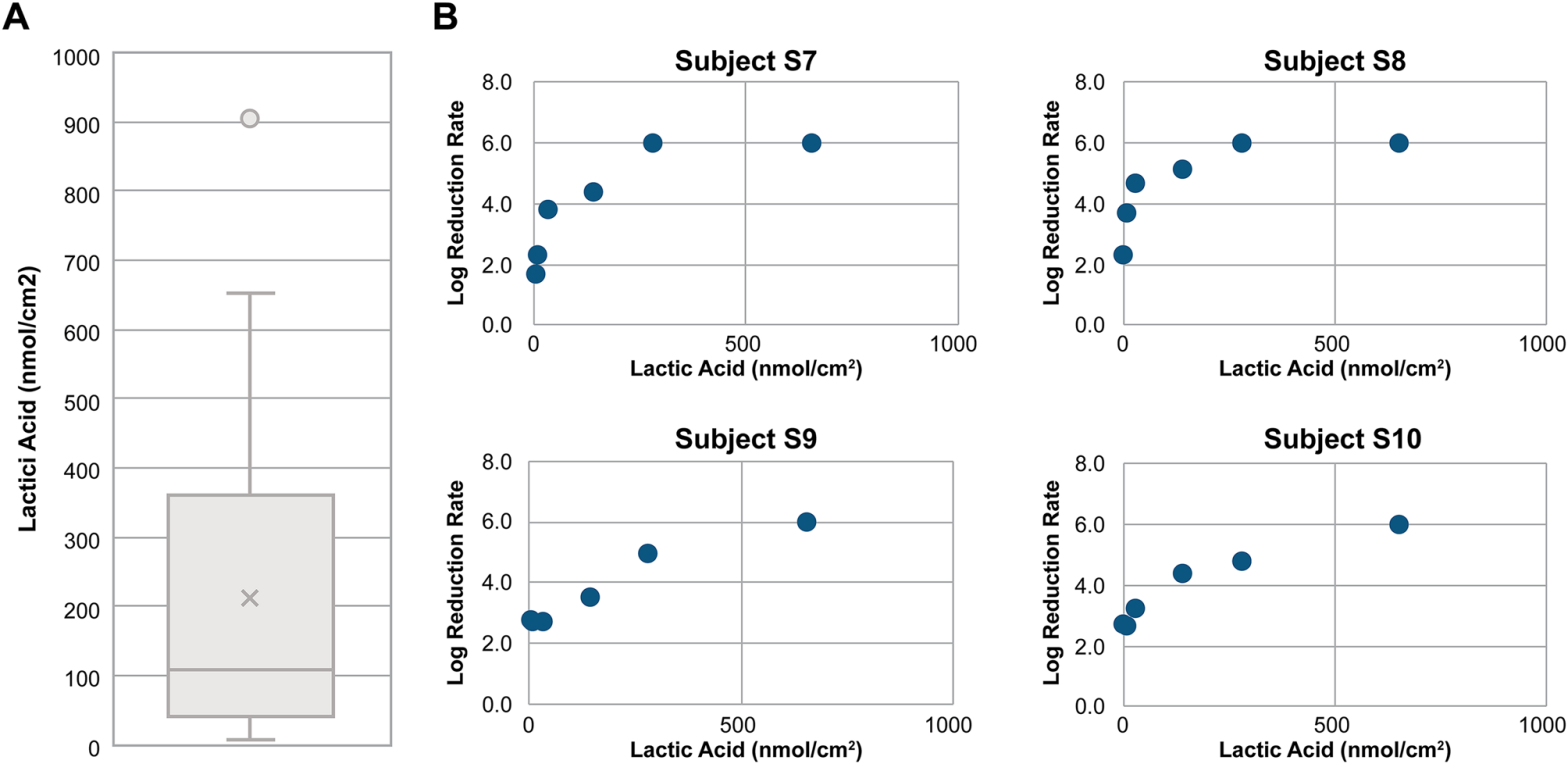
Verification of the effect of lactic acid on hand antimicrobial activity. (**A**) Amount of lactic acid on the surface of the hands of the participants (N = 54). (**B**) Bactericidal effect of applying lactic acid onto the hands of the participants. The test was made in a 2 cm × 2 cm area on the palm. After hand washing, 10 µL of 0.2, 1.0, 5.0, 10, and 23.5 g/L lactic acid aqueous solutions along with pure water as control were applied on the test site, followed by drying for 5 min. Then, 10 µl of the bacterial solution (*E. coli*, OD = 1.0) was added to the test site, spread for 30 s, and dried for 3 min. The amount of applied lactic acid on skin surface is plotted along X axis. The log reduction value denoting the relative logarithmic reduction of viable bacteria at each lactic acid amount and control is plotted along Y axis.

Lactic acid is abundant on the hands and is highly correlated with the antimicrobial activity of the surface layer component. Lactic acid has also been shown to have antimicrobial activity in test tube experiments^20^. However, these results are based on the evaluation of liquid contact, and it remains unclear whether the amount of lactic acid used in this study can enhance the levels of hand antimicrobial activity. Therefore, based on the amount of lactic acid found in 54 participants (Fig. 4A), a lactic acid solution was added to the hands of four randomly selected individuals (S7-S10) to verify whether it improved the antimicrobial activity (Supplementary Fig. S6). The participants washed their hands immediately before the experiment to reduce the contribution of natural lactic acid to the skin. Immediately after hand washing, we confirmed that the amount of lactic acid on the hands of each participant was < 30 nmoL/cm^2^ (Fig. 4B). Although individual differences were observed in all four participants, an improvement in antimicrobial activity was confirmed based on the amount of lactic acid on the hands, suggesting that lactic acid is important for hand antimicrobial activity. Furthermore, hydrochloric acid (HCl) solution with a pH value (pH = 2.24) equivalent to that of the maximum amount of lactic acid in Fig. 4B (652 mol/cm^2^) was applied to the hands of participants S8 and S10. HCl was also found to improve the antimicrobial activity (*P* < 0.05), although not as much as lactic acid (*P* < 0.05) (Supplementary Fig. S7).

## Discussion

Although washing hands using alcohol-based hand sanitizers removes/controls the number of surface microbes, the outcome is temporary. However, leveraging the innate defense of hands is more natural and permanent. Thus, we aimed to develop a hand hygiene maintenance method that leverages natural hand antimicrobial activity. In this study, we developed a novel methodology to quantify and characterize the function of the surface infection barrier on the hands that inactivates bacteria and viruses. For the first time, we demonstrated that the infection barrier found on the hand surface plays a crucial role in quantifying the risk of transmission of infectious diseases. In addition, we identified a list of components found on the hand surface that were correlated with antimicrobial activity and demonstrated that lactic acid is a key component of this infection barrier.

We confirmed the reliability of these methods by identifying a positive correlation between the in vitro and in vivo methods in which bacteria are applied to the hands. These data suggest that surface components are associated with hand antimicrobial activity. However, the correlation coefficient value was not high (r = 0.66, *P* = 0.1), suggesting that the hand antimicrobial activity is not only due to the surface components, but also to other factors, especially skin properties, such as pH and temperature^21,22^.

Interestingly, in this study, in addition to exhibiting higher hand antimicrobial activity, the low morbidity group performed hand washing and sanitizing less frequently than the high morbidity group. This may simply be due to greater hygiene awareness in the high morbidity group. Although hand washing and sanitizing are effective for the instantaneous removal of pathogens^23^, these actions cannot control the high frequency at which individuals unconsciously touch their face or potentially contaminated objects in their immediate environment. It is worth noting that the participants in this study, who were part of the general public, had less hygiene awareness than medical professionals. This suggests that inherent hand antimicrobial activity plays a role in preventing infections via unconscious transmission.

In addition, our study showed that both hand antimicrobial activity and oral mucosal moisture (and saliva volume) were associated with infection prevalence. Oral mucosal moisture is used as an objective indicator of the amount of saliva secretion^24^, and a similar tendency was observed in the actual saliva volume. Saliva protects against infections and is important in preventing attacks from pathogens^25,26^. The results of this study strongly suggest that both the hands and the oral cavity act as a first-line defense against bacterial and viral infections and thus are important for preventing infection. In contrast, there was no significant difference in NK cell activity and nasal mucosa clearance between the low morbidity and high morbidity groups. Many studies have shown that these biological functions are important for the prevention of infectious diseases^27–29^. Limiting the attributes of the participants, the two groups in this study had similar lifestyles, such as means of transportation, job type, sleep time, and stress level. Thus, the population studied is unlikely to exhibit differences in NK cell activity and nasal mucosa clearance. The small sample size was the main limitation of this retrospective study. Hence, large-scale and prospective trials are warranted to better clarify the association between hand antimicrobial activity and morbidity.

Next, a comprehensive analysis was performed to identify the factors that control hand surface barrier activity. In a previous study, sebum-containing components, especially fatty acids, on the skin were found to have antimicrobial properties^30^. However, none had a significant positive correlation with hand antimicrobial activity and only had a negative correlation with DAG. Compared with previous studies, the amount of lipids detected was relatively small; since there are no sebaceous glands in the palm, it is highly likely that the lipids analyzed were mainly derived from sources other than the hands. In contrast, prolactin-inducible protein was significantly correlated with antimicrobial activities among the water-soluble antimicrobial proteins and was involved in the surface infection barrier on the hands^31^. Although the detailed mechanism of the antimicrobial property of PRL is not yet fully understood, it binds to and aggregates the bacteria^32^. Furthermore, several amino acids were also positively correlated with antimicrobial activity; however, there are no prior reports related to their antimicrobial properties, and the details underlying this action remain unknown.

Lactic acid is prominently present in the hands. Although the hands lack sebaceous glands, they have a higher sweat gland density (518/cm^2^) compared to other sites^33^. Previous studies have documented various beneficial effects of lactic acid bacteria and their extracts on improving skin conditions, disease prevention, pH regulation, and depigmentation^34^. Although some studies have reported the presence of organic acids in sweat, there is a lack of research on the existence of natural lactic acid content on the human skin, especially on the hands, with a focus on antimicrobial activity^35,36^. In addition to lactic acid, several organic acids exert antimicrobial activity^37^. In general, organic acids are non-dissociative and pass through the cell membrane of the bacterium, before dissociating and releasing protons to damage the bacterium from within^38^. The antimicrobial properties of organic acids depend on their pH^20^. Interestingly, almost all organic acids that exhibit a significant correlation with antimicrobial activity are key metabolites of metabolic pathways (e.g., malic acid, fumaric acid, and citric acid). This suggests that, in addition to lowering the pH of the hands, their presence on the surface of hands may give rise to a metabolic environment that is not favorable for microbial proliferation. This is supported by the fact that lactic acid exhibits higher antimicrobial activity than an HCl solution with a similar pH.

Our study had a few limitations. First, as the study only included Japanese participants, the generalizability of the study to other racial and ethnic groups cannot be established. Second, the clinical survey that studied morbidity was retrospective in nature, and the participants were limited in terms of their living condition, with children in grade school. A prospective study with a larger sample size and including participants with varied living or demographic conditions would provide more meaningful insights. Third, the hand surface infection barrier could not be explained only by the surface component activity (Fig. 1B), suggesting that some skin properties such as pH, temperature, and water content might be involved in the antimicrobial activity.

In this study, we clarified that the surface infection barrier on the hands contributes to controlling the risk of infectious diseases, and lactic acid is suggested to be one of the crucial components of the hand surface infection barrier. These results could contribute to the development of a new natural antimicrobial hand hygiene technology with fewer side effects. However, the application of lactic acid solutions raises several issues: (1) skin damage, since it is an alpha hydroxylic acid and a highly acidic solution^39^; (2) variations in the pH (ranging from 4.0 to 7.0) of the skin among individuals, which would affect the efficacy of lactic acid against antimicrobial activity^40^; and (3) unknown long-lasting antimicrobial effects. Therefore, a more complex formulation will need to be identified to resolve these issues. In recent decades, the risk of infection by unseen pathogens has increased because of the dramatic population increase and the development of transportation networks. Thus, the development of a novel hand hygiene technology to enhance the antimicrobial properties of lactic acid on hands is worth studying to reduce the risk of unconscious contact infection in daily life.

## Methods

### Bacterial strains and culture medium

*E. coli* NBRC3301 strain (NBRC, National Institute of Technology and Evaluation Biological Resource Center) and *S. aureus* NBRC13276 strain were used and grown in Soybean-Casein Digest (SCD) agar medium and LB medium. The viable bacterial counts for antibacterial activity were measured by monitoring growth using a Bio Microplate Reader HiTS (Sinic Corporation). [Supplementary Methods].

### Viral strain, cells, and culture medium

Influenza virus A/Memphis/1/71 (H3N2) was propagated using Madin-Darby Canine Kidney (MDCK) cells in serum-free medium (SFM) (Supplementary Method), and viral titers were measured using focus-forming assays in MDCK cells^41^. The viral counts for antiviral activity were measured by determining the neuraminidase activity of the culture supernatant by slightly modifying the published methods[^42,43^, Supplementary Method].

### In vivo qualitative and quantitative evaluation of surface infection barrier on hands

The antimicrobial activity was measured twice in the same participant on different days using a hand stamp experiment for qualitative evaluation and a liquid cultivation method using a plate reader for quantitative evaluation (Supplementary Method).

### In vitro antimicrobial activity evaluation of hand surface components

To quantitatively measure the antimicrobial activity of the components found on the surface of the hands, we developed a novel, easy, and ethically favorable method that can be applied to many participants in a single survey period. A 2.5 cm × 5 cm region of the hand (Supplementary Fig. S2) was rubbed eight times with a swab 1K1501 (J.C.B. Industry Co.) soaked in 50% ethanol. This procedure was repeated thrice in the same region for each participant. Then, the swab tips were cut off and placed in a microtube, and 600 µL of 50% ethanol was added. The antibacterial effect was evaluated by calculating the number of surviving bacteria relative to the initial viable bacterial number. The antiviral effect was evaluated based on the number of residual viruses relative to the control virus number (Supplementary method).

### Blood sample collection and measurement of natural killer cell activity

Peripheral blood mononuclear cells (PBMCs) were isolated by density gradient centrifugation using Isolymph (CTL Scientific Supply Co.) and the natural killer (NK) cell activity of the PBMCs was determined using the chromium-51 (^51^Cr) release method^44^, Supplementary Methods).

### Saliva sample collection

Saliva samples were collected in a quiet room in the morning between 9:00 and 10:00, at least 60 min after eating. The participants were instructed to accumulate saliva in their mouths and spit it into a sterile plastic tube at the desired time. Saliva samples were collected for 10 min. The saliva volume (g) was calculated by subtracting the weight of the empty tube from that of the collected tube.

### Measurement of oral mucosal moisture and nasal mucociliary clearance

The oral mucosal moisture was collected on the same day of saliva collection and measured using an oral moisture-checking device (Mucus, Life Co.) according to the manufacturer’s instructions. The measurements were conducted thrice, and the mean values were calculated. The nasal mucociliary clearance time was evaluated using the saccharine test (15, 45, Supplementary Methods).

### Sample collection method for hand surface component analysis

For the collection of water-soluble components, nine circular pieces of φ2.1 cm filter paper (AS ONE Co.) were attached to the palm of the left hand for 5 min, and 60 µL of ultrapure water was added dropwise to each filter paper (Supplementary Fig. S4; Supplementary Methods).

### Analysis of organic acids and amino acids, water-soluble proteins, and lipids on the skin surface

Three filter papers sampled from the hand surface were dried by spraying with nitrogen. Subsequently, ultrapure water was added, and the supernatant was subjected to liquid chromatography–mass spectrometry^46^. Water-soluble proteins were dissolved in MPEX PTS Reagents (GL Sciences) containing 7 M urea and 2 M thiourea from six filter papers and subsequently identified^47,48^. Superficial lipids were quantified as previously described^49^. The details of these analyses can be found in the Supplementary Methods.

### Study design

We conducted four surveys to evaluate the surface infection barrier of the hand: (1) a preliminary study to understand the antimicrobial activity of hands using in vivo and in vitro methods, (2) a retrospective study investigating the relationship between antimicrobial activity of the hand and morbidity to infection, (3) the identification of antimicrobial components on hands using a comprehensive analysis, and (4) the verification of the effect of lactic acid application. We conducted surveys with different sets of participants. Patients with the following conditions were excluded from studies 3 and 4: (1) skin symptoms such as atopic dermatitis and rosacea-like dermatitis; (2) external wounds at the observation site; (3) previous allergic symptoms due to the use of external medicines, cosmetics, and quasi-drugs; (4) use of antibiotics or antifungal agents during the test or within the past month from the test date; and (5) pregnant or may be pregnant, and still within the postpartum period of less than 6 months. In study 2, patients with serious diseases (liver disorder, kidney disorder, myocardial infarction, etc.) and who regularly go to the hospital or take oral medicine for the purpose of treatment were also excluded. The participants of the abovementioned studies provided informed consent.

### Preliminary study to understand hand antimicrobial activity using in vivo and in vitro methods

To validate the feasibility of the method we developed, we randomly recruited healthy adult men and women (25–49 years old) as volunteers and conducted a pilot study in October 2020. The sampling sites are presented in Supplementary Fig. S1.

### Retrospective study investigating the relationship between the hand antimicrobial activity and morbidity to infection

We hypothesized that natural biological mechanisms, including hand antimicrobial activity and other physiological factors, differ between people susceptible and non-susceptible to infectious diseases. To test our hypothesis, we retrospectively studied the morbidity to infection of recruited participants in June 2018. Living with children below elementary school age is considered an environmental factor that greatly affects the prevalence of infectious diseases. Therefore, 28 healthy men and 71 women (aged 20–49 years) who lived with children under elementary school age were recruited. The sample size was determined according to a previous study^19^. Based on the responses to questionnaires on infectious diseases determined by the frequencies of colds symptoms and influenza virus infection (Table S1), we constructed (1) a “high morbidity” group (55 participants) and (2) a “low morbidity” group (54 participants) (Table S1) and compared the group characteristics (Table S2). Consequently, the two groups had similar ages, sex, and lifestyles (Tables S1 and S2). The samples were collected to evaluate oral moisture, nasal mucociliary clearance, and hand antimicrobial activity (Supplementary Fig. S3). The environment condition of the room was controlled under constant temperature (23.1 °C–25.8 °C, Median 24.9 °C) and humidity (49%-61%, Median 53%). The participants had the following restrictions: (1) avoiding excessive drinking, eating, and performing strenuous exercise the day before the test; (2) no smoking on the day of the test until the end of the test; (3) avoiding breakfast and brushing teeth for at least 1 h before starting the test; (4) drinking water only 1 h before starting the test; and (5) avoiding hand washing during the test.

In addition to measuring the biological parameters, a lifestyle-related questionnaire was also provided (Table S2). Statistical analysis was performed using the Statistical Package for the Social Sciences software for Windows. The data were first tested for normality using the Shapiro–Wilk test. Data showing normality were assessed for variability using an f-test and t-test, in accordance with body temperature, NK cell activity in PBMCs, and nasal mucociliary clearance. Non-normal data were tested using the Mann–Whitney U test (saliva volume, oral mucosa moisture, and hand antimicrobial activity for *E. coli* and *S. aureus*). In addition, a decision tree analysis was performed to assess the biological parameters (age, sex, and body temperature) and physiological functions (antimicrobial activity of hand, saliva volume, oral mucosal moisture, NK cell activity in PBMCs, and nasal mucociliary clearance) associated with morbidity and infection. In this analysis, we started with a core node consisting of the entire sample, and then recursively partitioned each node into two child nodes to create a tree-like structure. Odds ratios (ORs) and 95% confidence intervals (CIs) were obtained using the cross-tabulation method.

### Identification of antimicrobial components on hands using comprehensive analysis

To identify the factors involved in the antimicrobial components of the hands, we conducted an independent survey in January 2018. The following tests were performed: 1. analysis of organic acids and amino acids on the skin surface; 2. analysis of water-soluble proteins on the skin surface, and 3. analysis of lipids on the skin surface. The sample size was determined according to a previous study^30^. The study included 27 healthy men and 27 women (aged 20–49 years). The participants performed standard hand washing (50, Supplementary Methods) before hand-component collection (Supplementary Fig. S4). The correlation coefficient between each compound and antimicrobial activity was calculated using the correlation test function in R.

### Verification of the effect of lactic acid application on the hand antimicrobial activity

We recruited healthy individuals (aged 20–49 years) in September 2020 for this test. A 2 cm × 2 cm area of the participants’ palms was used as the test site (Supplementary Fig. S6; Supplementary Methods).

### Ethical approval

The study protocol, including sample collection, was reviewed and approved by the Ethical Committee of Kao Corporation. Study (1), a preliminary study to understand the hand antimicrobial activity using in vivo and in vitro methods, and study (4), verification of the effect of lactic acid application on the hand antimicrobial activity, were approved as S311-200,807; study (2), a retrospective study investigating the relationship between hand antimicrobial activity and morbidity to infection, was approved as T116-18,032, and study (3), identification of antimicrobial components on hands using comprehensive analysis, was approved as S108-171,024. Informed written consent was obtained from all participants after the procedures had been explained, and documentation was performed. All experiments were conducted in accordance with the principles of the Declaration of Helsinki.

## Supplementary Information

Supplementary Information.

## Data Availability

All data generated or analyzed during this study are included in this published article (and its Supplementary Information files).
