# Supplementary material for: Lactic acid as a major contributor to hand surface infection barrier and its association with morbidity to infectious disease
Source: Sci Rep. 2021 Sep 20;11:18608. doi: 10.1038/s41598-021-98042-4 (PMC8452697; doi:10.1038/s41598-021-98042-4)
Supplement: Supplementary file 1 — Supplementary Information. [file 41598_2021_98042_MOESM1_ESM.pdf]

Lactic acid as a major contributor to hand surface infection barrier and its association with morbidity to infectious disease

Yuki Nishioka,[a] Kenichi Nagano,[b] Yoshitaka Koga,[c] Yasuhiro Okada,[a] Ichiro Mori,[a] Atsuko Hayase,[c] Takuya Mori,[c] and Kenji Manabe[a),*]

[a]Personal Health Care Products Research, Kao Corporation, 2-1-3, Bunka, Sumida-ku, Tokyo 131-8501, Japan

[b]Biological Science Laboratories, Kao Corporation, 2606 Akabane, Ichikai, Haga, Tochigi, 321-3497, Japan

[c]Analytical Science Laboratories, Kao Corporation, 2606 Akabane, Ichikai, Haga, Tochigi, 321-3497, Japan

***Corresponding author:**

Kao Corporation, 2-1-3, Bunka, Sumida-ku, Tokyo 131-8501, Japan

Tel: +81-3-5630-9461

Fax: +81-3-5630-9647

E-mail: manabe.kenji@kao.com

Supplementary Information

Supplementary methods

Bacterial strains and culture medium

Escherichia coli NBRC3301 strain (NBRC, National Institute of Technology and Evaluation Biological Resource Center) and *Staphylococcus aureus* NBRC13276 strain were used and grown as described in the Supplementary Methods. As a pre-culture, a single colony was grown on Soybean-Casein Digest (SCD) agar medium (Nihon Pharmaceutical. Co., Ltd), and then inoculated into 4 mL of Luria-Bertani (LB) liquid medium (Nihon Pharmaceutical. Co., Ltd) and cultured overnight at 37°C and 180 rpm. Next, 1% of the obtained culture solution was inoculated into an LB liquid medium, cultured for 15 h, washed twice with sterile water, and stored on ice.

Viral strain, cells, and culture medium

Madin-Darby canine kidney (MDCK) cells (ATCC CCL-34) were cultured in minimum essential medium (MEM; Invitrogen Corporation, NY) supplemented with 5% fetal bovine serum (Sigma-Aldrich Co.) and 50 μg/mL gentamicin (Invitrogen Corporation). Influenza virus A/Memphis/1/71 (H3N2) was propagated using MDCK cells in serum-free medium (SFM) (Thermo Fisher Scientific) supplemented with 2 μg/mL acetylated

trypsin (Sigma-Aldrich) and 50 μg/mL gentamicin. The virus was purified by centrifugation at 13,000×g for 2 h and resuspended in SFM. Viral titers were measured using focus-forming assays.

Measurement of viable bacterial counts for antibacterial activity by the culture method using a plate reader

The viable bacterial counts for antibacterial activity were measured by monitoring growth using a Bio Microplate Reader HiTS (Sinic Corporation). A dilution series was prepared from the bacterial suspensions, and the bacterial numbers were measured by plating on an SCD agar medium and incubating for 18 h at 37°C. Then, the sample containing bacteria and the dilution series were shaken at 37°C. The absorbance (OD600) was measured every 15 min to generate a growth curve. Based on the resulting growth curve, the time required to reach OD600 = 0.02, which is the middle stage of the logarithmic growth phase, was obtained, and the number of viable bacteria in the sample was calculated using the calibration curve obtained from the dilution series.

Measurement of viral counts for antiviral activity using neuraminidase activity assay

The viral counts for antiviral activity were measured by determining the neuraminidase activity of the culture supernatant based on the methods used in previous studies. First, a sample solution containing viruses was added to MDCK cells in a 96-well plate and incubated for 15 min at 37°C under 5% CO2. The MDCK cells were then washed twice with 100 µL of phosphate-buffered saline (PBS) (FUJIFILM Wako Pure Chemical Co.). The MDCK cells were then incubated at 37°C under a 5% CO2 atmosphere in 100 µL of SFM for 14 h, and the amount of propagated virus was calculated based on the level of neuraminidase activity in the supernatant. For the neuraminidase activity assay, 20 µL of 250 µM 4-methylumbelliferyl N-acetyl-α-D-neuraminic acid (Funakoshi Co.) was added to 30 µL of the supernatant and incubated at 37°C for 30 min. The reaction was terminated by adding 200 µL of 100 mM sodium carbonate buffer, and the fluorescence value was measured using a plate reader (Ex: 355 nm/Em: 460 nm, gain: 70) (TECAN M200). The number of viruses in the sample solution was calculated from a calibration curve obtained from the dilution series of the virus suspension counted in advance using focus-forming assays.

In vivo qualitative and quantitative evaluation of surface infection barrier on hands

For qualitative evaluation, *E. coli* solution (200 mL) with an optical density (OD) of 0.1, was placed in a 500 mL vat. Both hands were soaked for 5 s, and the left hand was dried for 3 min. Residual bacteria were qualitatively observed by placing the hand on a Petri dish (Nissui Pharmaceutical Co., Ltd.) containing X-Gal agar medium (Nissui Pharmaceutical Co., Ltd.) for 10 s (loading: 2 kg) and culturing in a 37°C incubator for 15 h. The other hand was stamped after drying for 30 s as a control. Blue colonies indicate that *E. coli* degraded X-gal in the medium.

For quantitative evaluation, 10 µL of cultured *E. coli* solution (OD = 1.0) was applied to a 4 cm^2 area of the palm, and was collected 3 min later using a swab (BD-BBL culture swab EZ; Becton Dickinson) soaked in physiological saline. Collection by swabbing was performed twice, and the pooled sample was incubated in 1 mL of lecithin and polysorbate 80 (LP) medium (FUJIFILM Wako Pure Chemical Co.). One hundred microliters of this sample was mixed with 100 µL of 2× SCD medium (Nihon Pharmaceutical Co., Ltd.) and cultivated in a 96-well microplate in a 37°C incubator for 24 h. Quantification was performed using a microplate reader (Bio Microplate Reader HiTS; Sinic Corporation), and the antimicrobial effect was evaluated by calculating the number of surviving bacteria relative to the initial viable bacterial number.

Measurement of nasal mucociliary clearance

The nasal mucociliary clearance time was evaluated using the saccharine test. A tablet was placed in the nasal cavity of the participants, who were instructed to settle in a comfortable sitting position and to avoid sneezing, coughing, sniffling, blowing their nose, talking, or taking deep breaths during the test. The time taken from the placement of the tablet to the perception of sweet taste was recorded as the nasal mucociliary clearance time.

Sample collection method for hand surface component analysis

The filter paper was divided into three sheets (for low-molecular-weight compounds) and six sheets (for antimicrobial peptides), and each was collected in a screw tube. For lipid collection on the skin surface, a 5 cm × 5 cm area of the left palm was scraped with a swab soaked in ethanol (three times from the same site) and collected in a screw tube.

Analysis of water-soluble proteins on the skin surface

Water-soluble proteins were dissolved in MPEX PTS Reagents (GL Sciences) containing 7 M urea and 2 M thiourea from six filter papers. Proteins were identified as described

previously. Proteins were identified by cross-referencing the obtained spectrum with the Swiss-Prot protein sequence database. The concentrations of the identified proteins were estimated using the emPAI method.

Analysis of lipids on the skin surface

Superficial lipids were quantified as previously described. After immersing a cotton swab with scraped hands in chloroform/methanol (1/1 solution), the supernatant was collected and internal standard substances (TAG-C39: 0: tritridecanoin, DAG-C26: 0: ditridecanoin, FFA-C12: 0- d3: lauric acid (Methyl-d3), WE-C28: 1: lauryl palmitoleate, ChE-C10: 0: cholesteryl caprate, ChE-C2: 0: cholesteryl acetate) were added to the samples, which were then subjected to liquid chromatography tandem-mass spectrometry. An Agilent 1200 series LC system equipped with an Agilent 6460 set ESI source was used.

In vitro antimicrobial activity evaluation of hand surface components

The samples were sonicated for 5 min using an ultrasonic cleaner SW5800 (CITIZEN Watch Co.) and pooled into one microtube (1800 µL per subject). After centrifugation at 15,000 rpm for 5 min, the supernatant was split into 2 to evaluate antibacterial and

antiviral activity. Then, the samples were dried using a centrifugal evaporator CVE-3000 (TOKYO RIKAKIKAI CO, LTD), dissolved in 10 µL of dimethyl sulfoxide (DMSO) (FUJIFILM Wako Pure Chemical Co.), and stored at −20°C. For antibacterial activity, an undiluted solution of surface components on the hands was used against *E. coli*, and an eight-fold diluted sample with PBS was used against *S. aureus*. The sample and the bacterial solution (OD600 = 1.0) were mixed in equal amounts and reacted at 37°C for 1 h. Quenching was performed by adding 135 µL of lecithin and polysorbate 80 (LP) diluent to 15 µL of the reaction solution and then was placed on ice. Next, 100 µL of this sample was mixed with 100 µL of 2 × SCD medium and cultivated in a 96-well microplate in a 37°C incubator for 24 h, before quantification was performed on a microplate reader (Bio Microplate Reader HiTS; Sinic Corporation). The antibacterial effect was evaluated by calculating the number of surviving bacteria relative to the initial viable bacterial number. For antiviral evaluation, surface components extracted from the hand were diluted 100-fold with PBS, and 100 µL was incubated with 100 µL of virus solution (8.0 × 104 pfu/mL) at 37°C for 30 min, in accordance with our standard laboratory protocol. After the reaction, 100 µL of serum-free medium (SFM) at a double concentration was added and placed on ice for quenching. The antiviral effect was evaluated based on the number of residual viruses relative to the control virus number.

Blood sample collection and measurement of natural killer cell activity

Blood samples were collected in heparinized tubes using a lymphocyte preservation solution (Nipro Co.). Peripheral blood mononuclear cells (PBMCs) were isolated by density gradient centrifugation using Isolymph (CTL Scientific Supply Co.), according to the manufacturer's instructions. The natural killer (NK) cell activity of PBMCs was determined using the chromium-51 (51Cr) release method. Briefly, PBMCs isolated as effector cells and K-562 tumor cells labeled with 51Cr as target cells were incubated at a ratio of 1:50 for 4 h at 37°C. Then, the 51Cr released in the supernatant was measured using a γ-counter (ARC-370M, Aloka). The maximum 51Cr release was ensured by adding 1 N hydrochloric acid, and the minimum 51Cr release was confirmed by adding only complete medium to the target cells. NK cell activity was expressed as the percentage of cytotoxicity, calculated using the following formula:

NK cell activity = [(experiment cpm – minimum cpm)/(maximum cpm – minimum cpm)] × 100.

Preliminary study to understand the antimicrobial activity of on hands using *in vivo* and *in vitro* methods

The participants performed standard hand washing using an experimental soap formulated with alkyl ether carboxylic acid, sodium salt (AEC), alkyl ether sulfate (AES), and alkyl glucoside (AG) without antimicrobial compounds [50], rinsed with tap water for 30 s, and then washed with purified water for 10 seconds. To avoid contact with the evaluation site, they wore polyethylene gloves (AS ONE Co.) for 2 h of acclimation. Subsequently, qualitative and quantitative evaluations of hand antimicrobial ability were performed according to the method described separately.

Identification of antimicrobial components on hands using comprehensive analysis

The participants performed standard hand washing with test using an experimental soap formulated with AEC, AES, and AG without antimicrobial compounds, rinsed with tap water for 30 s, and then washed with purified water for 10 seconds. To avoid contact with the evaluation site, they wore polyethylene gloves (0950; SHOWA GLOVE Co.) for 2 h of acclimation (20°C, 40% humidity).

Verification of the effect of lactic acid application on hand antimicrobial activity

After hand washing, 10 µL of 0.2, 1.0, 5.0, 10, and 23.5 g/L lactic acid aqueous solution (FUJIFILM Wako Pure Chemical Corporation) was added dropwise to the test site, spread over 30 s using a 0.2 mL round tube (AS ONE Co.), and dried for 5 min. During this process, 5.6, 27.8, 138.8, 277.5, and 652.5 nmoL/cm^2 of lactic acid remained on the hands, respectively. Next, 10 µL of bacterial solution (*E. coli*, OD = 1) was added dropwise to the test site, spread for 30 s using a 0.2 mL round tube, and dried for 3 min. The test site was scraped with a swab immersed in LP-PBS containing PBS with LP solution to collect the residual bacteria. Collection by swabbing was performed twice, and the collected samples were suspended in 1 mL of LP-PBS solution. Then, 100 µL of this sample was mixed with 100 µL of 2× SCD medium (Nihon Pharmaceutical Co.) and cultivated in a 96-well microplate in a 37°C incubator for 24 h, followed by quantification on a microplate reader (Bio Microplate Reader HiTS; Sinic Corporation). To determine whether the effect of lactic acid is mediated by pH, we used aqueous hydrochloric acid (HCl) solution (Kanto Chemical Co., Inc.), whose pH was adjusted to the value of lactic acid as the control.

Supplementary Data

Table S1. Participant characteristics

**Characteristics**	**High morbidity group**	**Low morbidity group**
n	55	54
Age (years), mean (SD)	39.9 (4.9)	40.6 (5.4)
Sex, n (%)		
Men	17	21
Women	38	33
Frequency of developing flu within 3 years (Positive test at medical institution[1])	Two times or more	0 times
Frequency of developing colds within a year (cough, runny nose, sore throat, etc. with fever of 37.5°C or higher)	Three times or more	0 times

[1]The flu was diagnosed at the medical institution as per the following criteria:

(1) Positive diagnosis result by a rapid influenza diagnostic test.

(2) Flu-like symptoms including sudden appearance of fever, high fever (37.5°C or more), symptoms of upper respiratory tract infection such as cough, runny nose, sore throat, etc., body aches

Table S2. Questionnaire regarding lifestyles and hygiene behaviors

Questionnaire	Answer	Low morbidity group (N=54)		High morbidity group (N=55)		*P*-value[1]
		Numbers[1]	Percentage[1]	Numbers[1]	Percentage[1]	
A. Regarding Lifestyles						
1) Are you commuting by train or bus?	Yes	22	41%	29	53%	0.210
	No	32	59%	26	47%	0.210
2) Do you often use public facilities (stations, libraries, movie theaters, sports facilities, parks, etc.) (twice or more/week)?	Yes	29	54%	29	53%	0.919
	No	25	46%	26	47%	0.919
3) Do you have a lot of desk work (5 hours or more/day)	Yes	22	41%	17	31%	0.284
	No	32	59%	38	69%	0.284
4) Do you smoke?	Smoking	7	13%	6	11%	0.741
	I smoked in the past	12	22%	18	33%	0.741
	I have never smoked	35	65%	31	56%	0.367
5) How often do you drink alcohol?	Almost every day	9	17%	4	7%	0.130
	within 5 days/week	9	17%	11	20%	0.653
	within 2 days/week	14	26%	16	29%	0.711
	Almost never drink	22	41%	24	44%	0.760
6) How often do you exercise for 30 minutes or more at a time	Almost every day	6	11%	14	25%	0.053
	within 5 days/week	2	4%	**10**	**18%**	**0.016**
	within 2 days/week	19	35%	14	25%	0.269

(including commuting such as walking or biking)	Almost never exercise	**27**	**50%**	17	31%	**0.042**
7) Do you have enough sleep	Yes	40	74%	37	67%	0.436
	No	14	26%	18	33%	0.436
8) Do you try to have a nutritionally balanced diet?	Yes	42	78%	47	85%	0.301
	No	12	22%	8	15%	0.301
9) Are you able to release stress?	Yes	37	69%	32	58%	0.263
	No	17	31%	23	42%	0.263
B. Regarding Hygiene Behaviors						
10) How often do you wash your hands?	Almost every day	44	81%	**53**	**96%**	0.013
	Sometimes (only during infection season)	7	13%	2	4%	0.077
	Nothing	3	6%	0	0%	0.077
11) How often do you use hand sanitizer?	Almost every day	1	2%	**9**	**16%**	**0.009**
	Sometimes (only during infection season)	24	44%	34	62%	0.069
	Nothing	**29**	**54%**	12	22%	**0.001**
12) How often do you gargle?	Almost every day	25	46%	**41**	**75%**	**0.003**
	Sometimes (only during infection season)	**24**	**44%**	12	22%	0.012
	Nothing	5	9%	2	4%	0.231
13) How often do you wear mask?	Almost every day	2	4%	7	13%	0.087
	Sometimes (only during infection season)	35	65%	**46**	**84%**	**0.025**
	Nothing	**17**	**31%**	2	4%	**0.000**
14) How much do you get the flu vaccine?	Two times every year	0	0%	2	4%	0.157
	One time every year	0	0%	**32**	**59%**	**0.000**

	Once every 2 years	0	0%	**8**	**15%**	**0.000**
	I have not been vaccinated in the last few years	**29**	**54%**	5	9%	**0.000**
	I have never / do not remember being vaccinated	**25**	**46%**	8	15%	**0.000**

[1]The significance test was performed with the null hypothesis. Bold numbers indicate significantly high values (*P*-value less than 0.05).

Table S3. Results calculated by the decision tree analysis

Parameters	Reference value calculated by the decision tree analysis	*P*-value	OR	95% CI
Antimicrobial activity of hand for *E. coli*	0.154	<0.001	7.2	2.64–19.50
oral mucosal moisture	27.7	0.004	5.7	2.08–15.60
age	41	0.033	0.12	0.01–1.08
	38	0.209	2.86	0.53–15.41

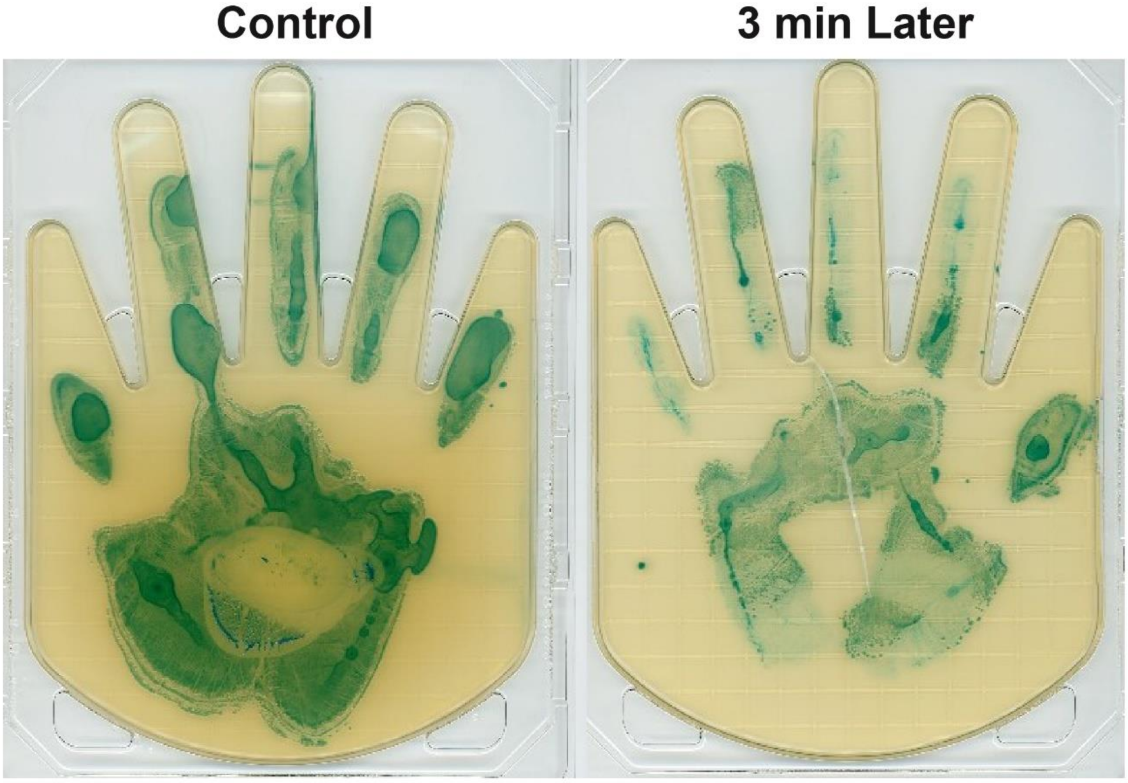

Figure S1. Results immediately after washing the hands of a person with high antimicrobial activity (Figure 1A left)

The results show 30 s as the control and 3 min after applying *E. coli* solution of OD = 0.2, on the hand.

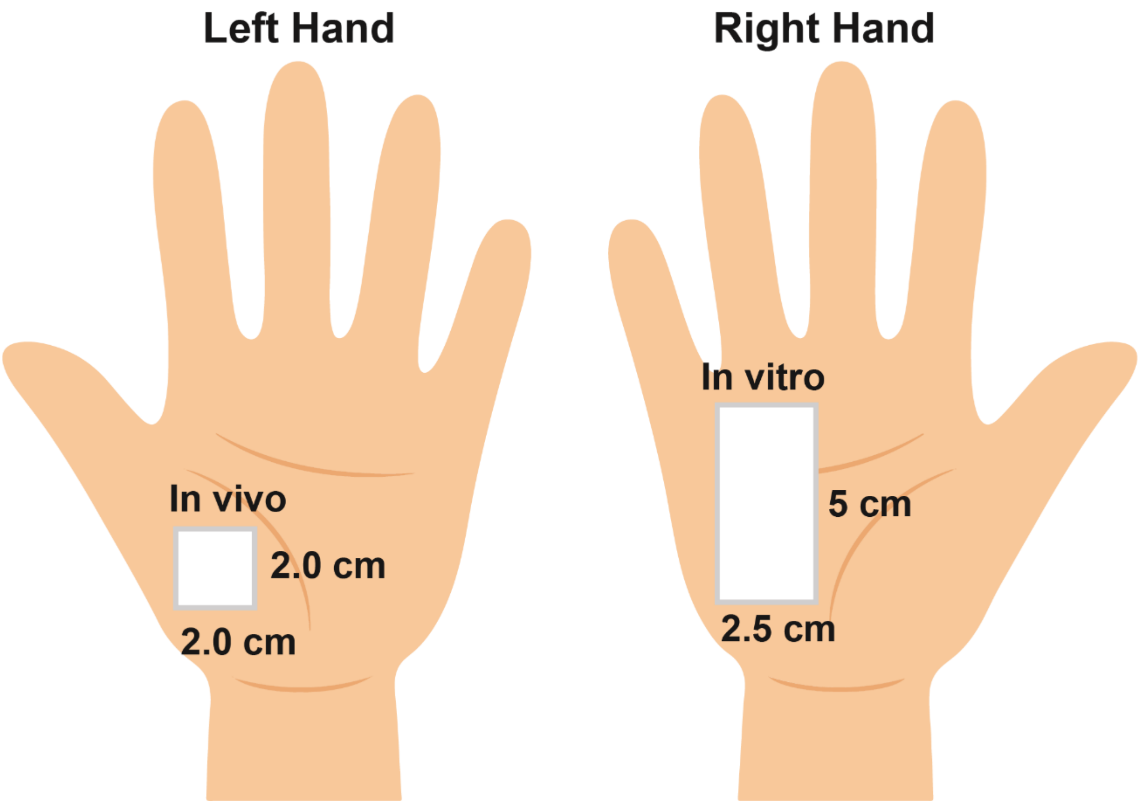

Figure S2. Sites for hand surface antimicrobial evaluation (*in vivo,* left hand) and sites for the collection of surface components for antimicrobial evaluation (*in vitro*, right hand)

For evaluation of surface infection barrier on hands, 10 µL of cultured *E. coli s*uspension (OD = 1.0) was applied to the 2.0 cm × 2.0 cm area of the palm of the left hand and was collected 3 min later using a swab soaked in physiological saline. To evaluate the antimicrobial activity of the surface components, a 2.5 cm × 5 cm area of the palm of the right hand was rubbed with a swab soaked in 50% ethanol and repeated three times at the same site.

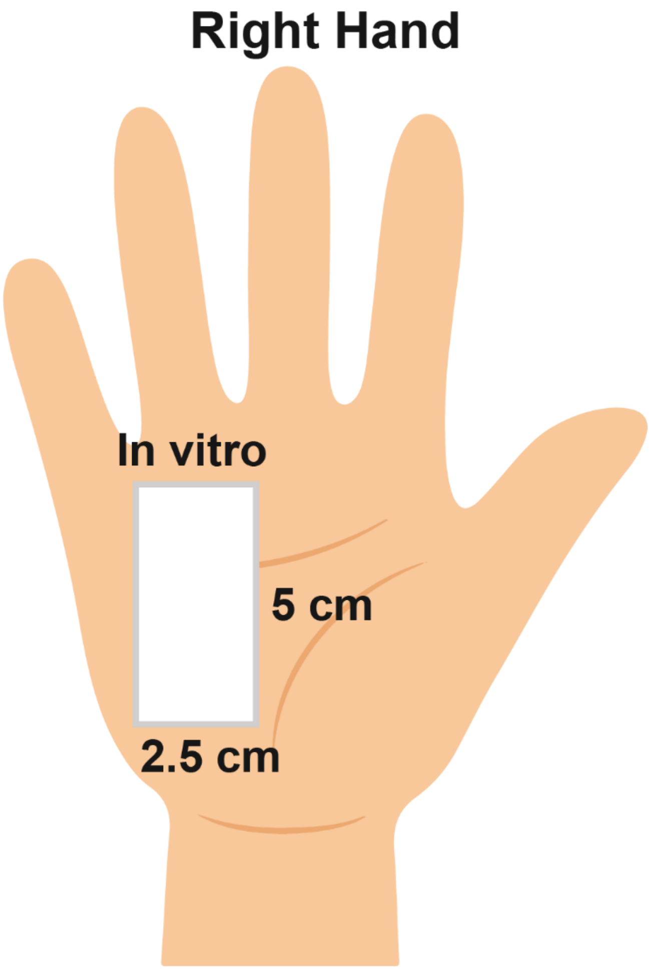

Figure S3. Sites for the collection of surface components for antimicrobial evaluation (*in vitro*, right hand)

To evaluate the antimicrobial activity of the surface components, a 2.5 × 5 cm area of the palm of the right hand was rubbed with a swab soaked in 50% ethanol and repeated three times at the same site.

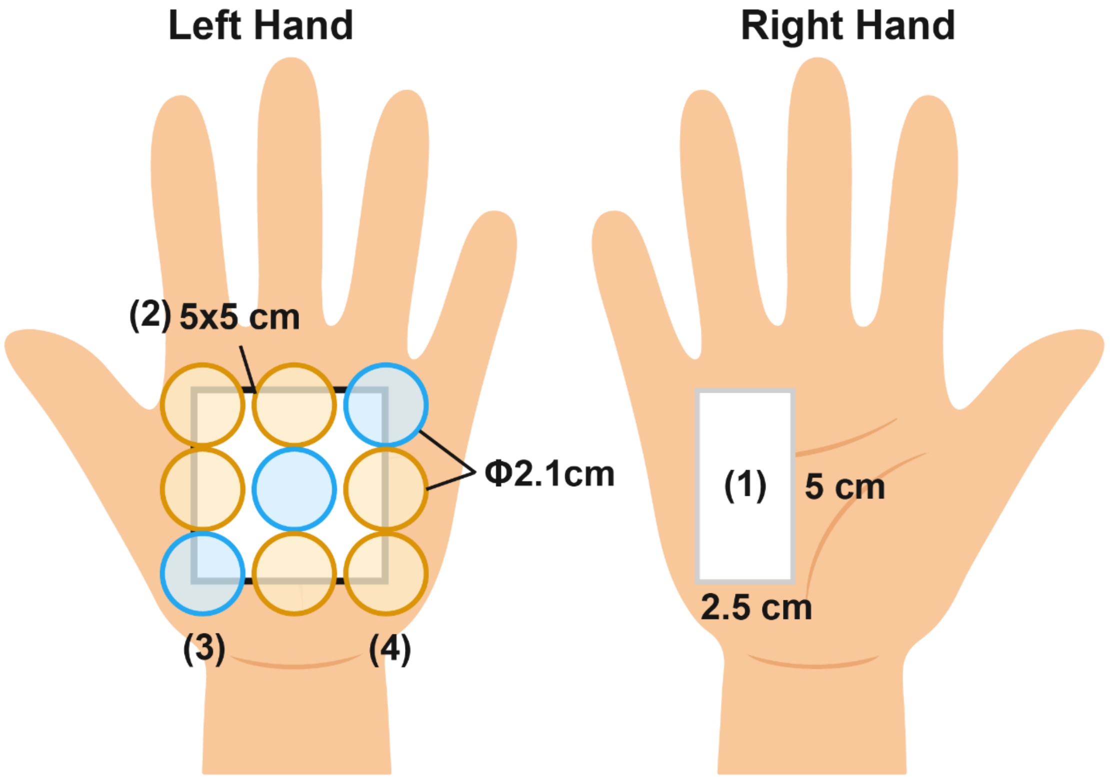

Figure S4. Sites for the collection of surface components for compound analysis (left hand) and antimicrobial analysis (right hand)

For antimicrobial analysis, the palm of the right hand (1) was rubbed with a swab soaked in 50% ethanol. For lipid collection on the skin surface, the left palm (2) was scraped with a swab soaked in ethanol and collected in a screw tube. For the collection of water-soluble components, nine sheets of filter paper were attached to the palm of the left hand for 5 min, and 60 μL of ultrapure water was added to each filter paper. The filter paper was divided into three sheets (3) for organic acids and amino acids, and 6 sheets (4) for antibacterial peptides.

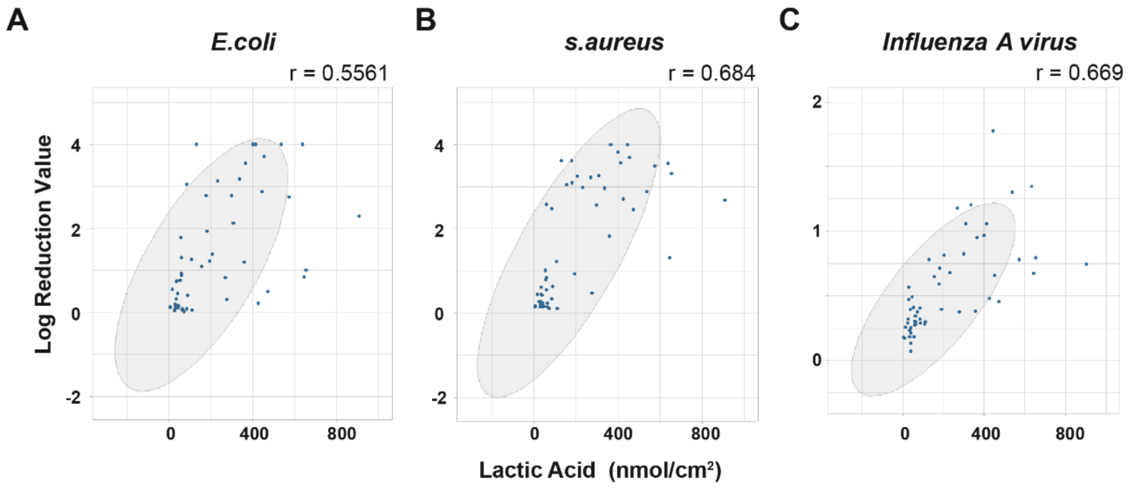

Figure S5. Relationship between the amount of lactic acid on hand surface and the antimicrobial activities of hand surface components

The horizontal axis shows the amount of lactic acid on the hands, and the vertical axis shows the antimicrobial activity. Antimicrobial activities were measured using *an in vitro* method, and the log reduction value indicated the relative logarithmic reduction of bacterial or viral numbers. A, B, and C show the results for *E. coli*, *S. aureus,* and influenza A virus.

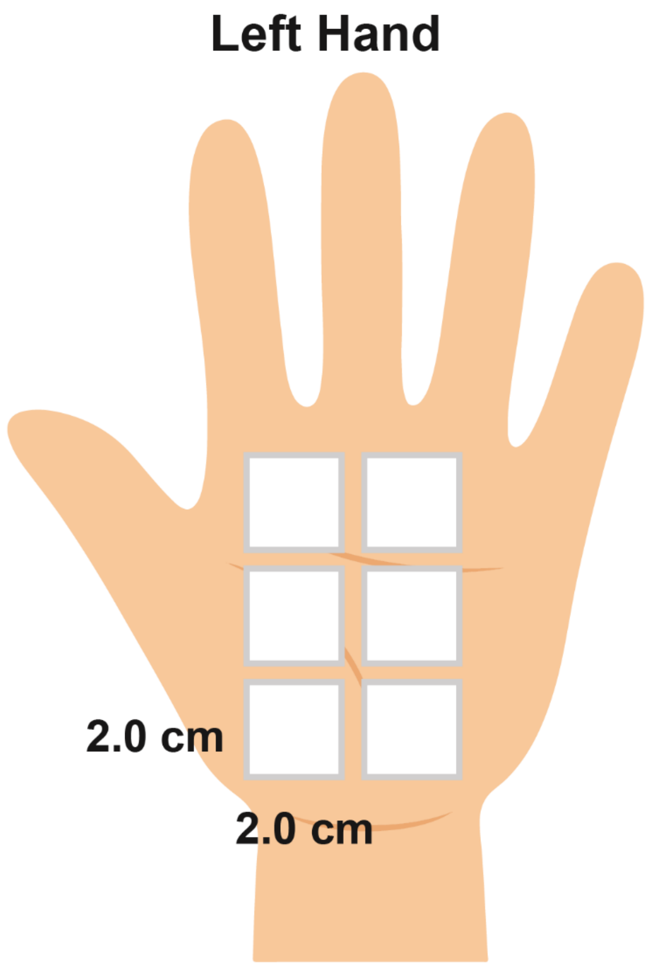

Figure S6. Sites for verification of the antibacterial effect of lactic acid application on hands

To verify the antibacterial effect of lactic acid application on hands, 10 µL of 0.2, 1.0, 5.0, 10, and 23.5 g/L lactic acid aqueous solution and pure water were applied to a 2.0 × 2.0 cm area of the palm from top to bottom. The samples were spread for 30 s and dried for 5 min. Approximately 10 µL of cultured *E. coli* suspension (OD = 1.0) was applied to the site and collected 3 min later using a swab soaked in physiological saline.

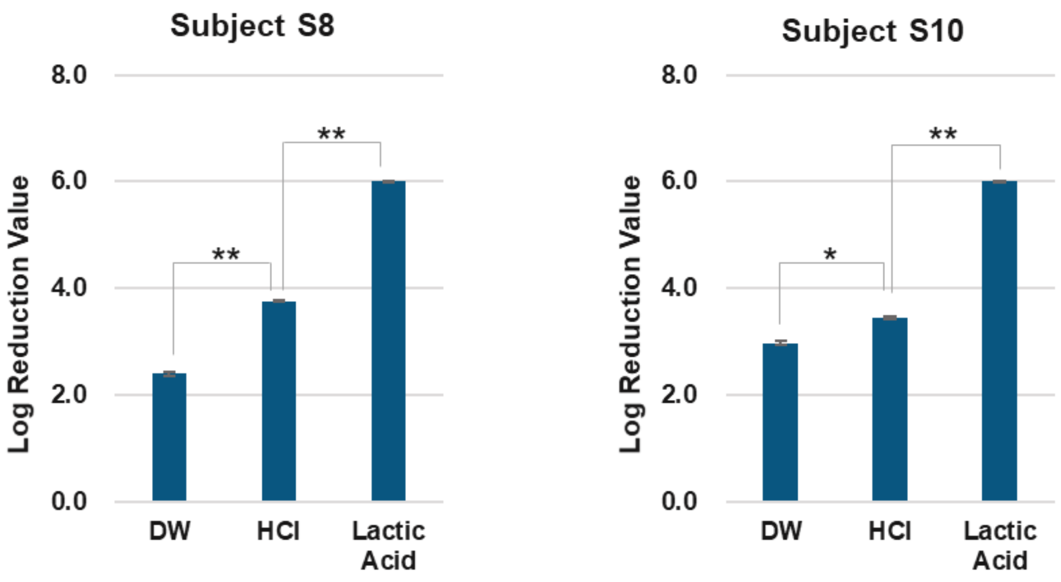

Figure S7. Comparison of hand bactericidal activities by application of hydrochloric acid solution with the same pH as lactic acid

An aqueous solution of lactic acid or hydrochloric acid (HCl) was added to the test site, spread, and dried. Next, a bacterial solution (*E. coli*, OD = 1.0) was added to the test site, spread, and dried. The log reduction value indicates a relative logarithmic reduction of viable bacteria. HCl was adjusted to 2.24, with the same pH as that of the lactic acid aqueous solution. Asterisks indicate a significant difference between two samples (t-test, **P<0.01, and *P<0.05).
